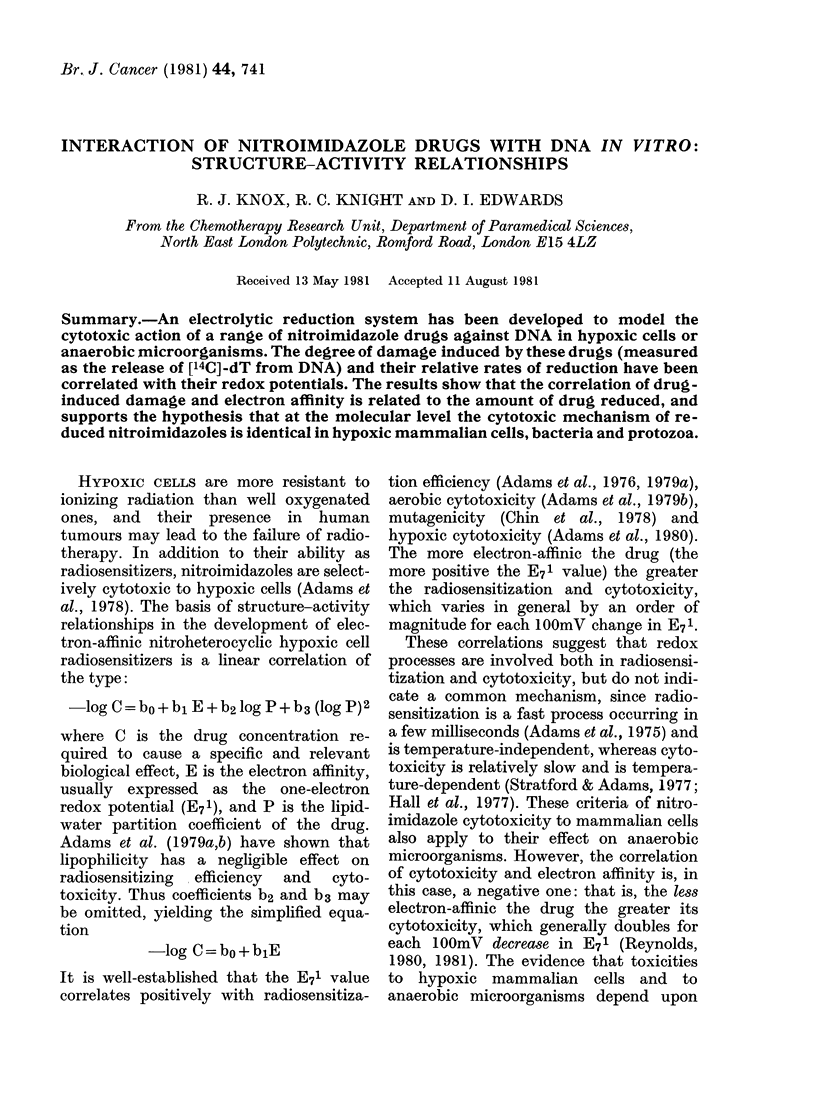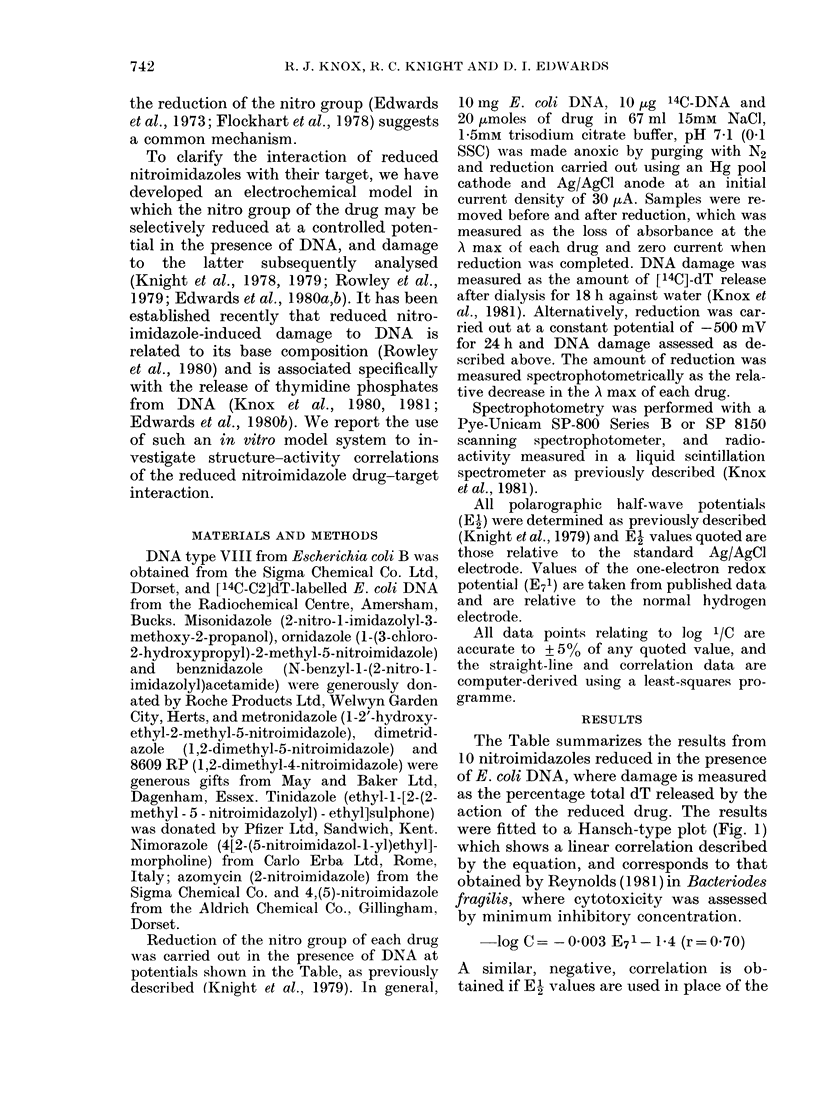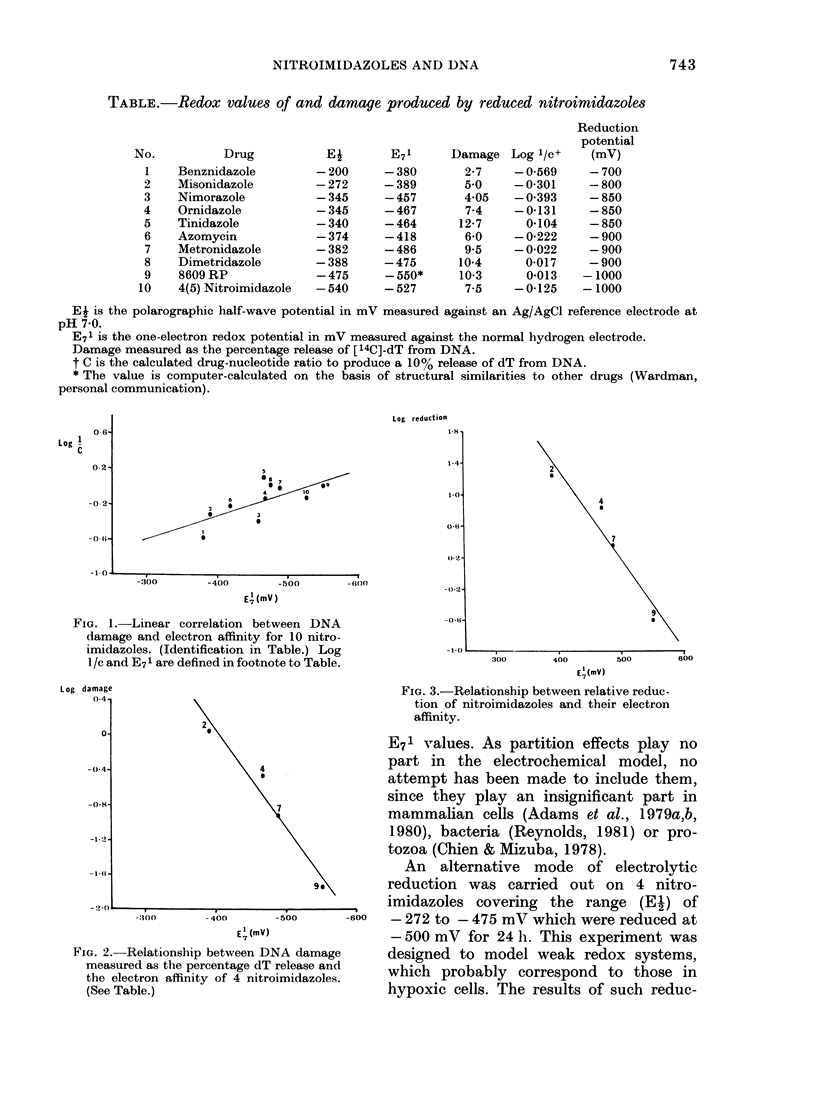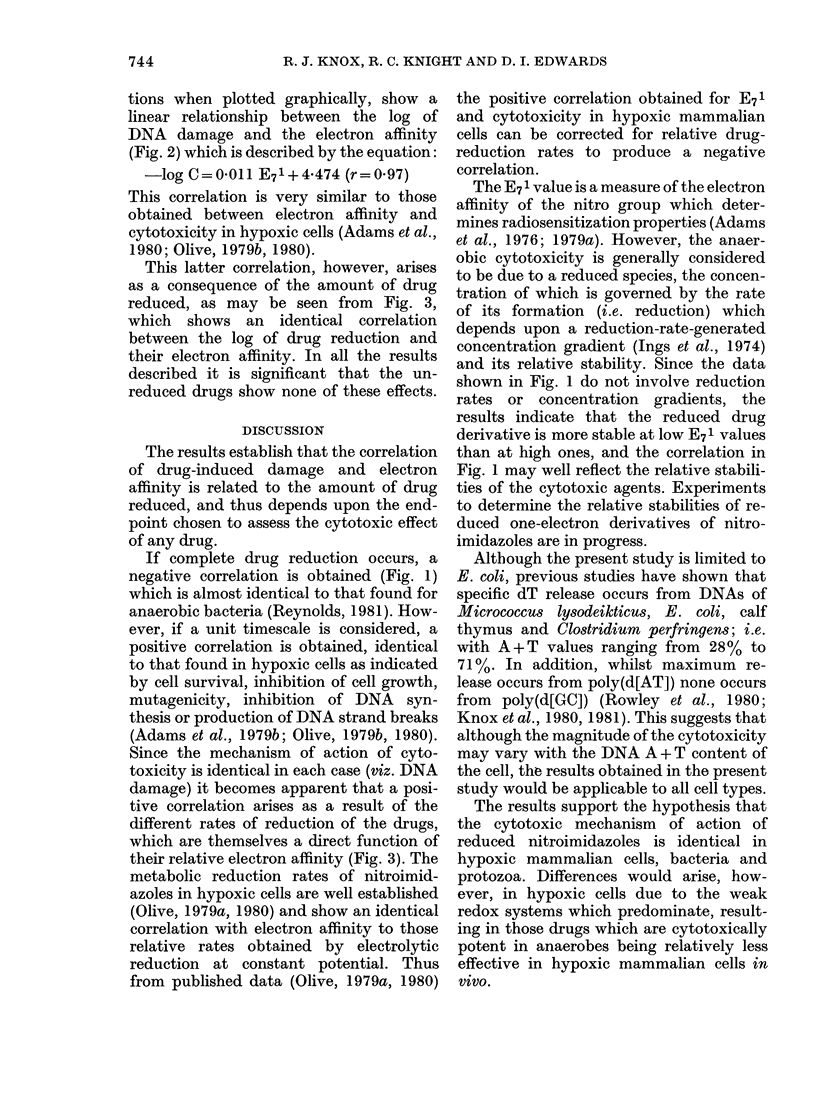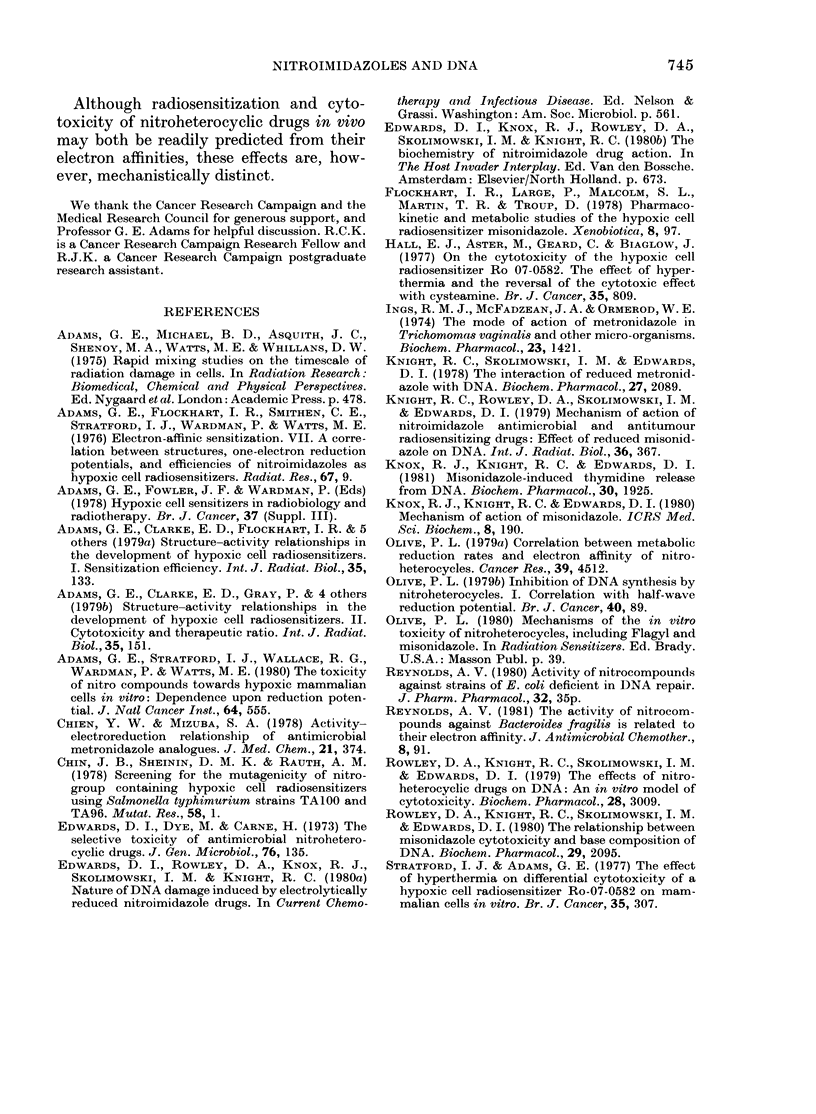# Interaction of nitroimidazole drugs with DNA in vitro: structure-activity relationships.

**DOI:** 10.1038/bjc.1981.261

**Published:** 1981-11

**Authors:** R. J. Knox, R. C. Knight, D. I. Edwards

## Abstract

An electrolytic reduction system has been developed to model the cytotoxic action of a range of nitroimidazole drugs against DNA hypoxic cells or anaerobic microorganisms. THe degree of damage induced by these drugs (measured as the release of [14C]-dT from DNA) and their relative rates of reduction have been correlated with their redox potentials. The results show that the correlation of drug-induced damage and electron affinity is related to the amount of drug reduced, and supports the hypothesis that at the molecular level the cytotoxic mechanism of reduced nitroimidazoles is identical in hypoxic mammalian cells, bacteria and protozoa.


Br. J. Cancer (1981) 44, 741

### INTERACTION OF NITROIMIDAZOLE DRUGS WITH DNA *IN VITRO*: STRUCTURE–ACTIVITY RELATIONSHIPS

R. J. KNOX, R. C. KNIGHT AND D. I. EDWARDS

*From the Chemotherapy Research Unit, Department of Paramedical Sciences, North East London Polytechnic, Romford Road, London E15 4LZ*

Received 13 May 1981 Accepted 11 August 1981

**Summary.—An electrolytic reduction system has been developed to model the cytotoxic action of a range of nitroimidazole drugs against DNA in hypoxic cells or anaerobic microorganisms. The degree of damage induced by these drugs (measured as the release of [$^{14}$C]-dT from DNA) and their relative rates of reduction have been correlated with their redox potentials. The results show that the correlation of drug-induced damage and electron affinity is related to the amount of drug reduced, and supports the hypothesis that at the molecular level the cytotoxic mechanism of reduced nitroimidazoles is identical in hypoxic mammalian cells, bacteria and protozoa.**

HYPOXIC CELLS are more resistant to ionizing radiation than well oxygenated ones, and their presence in human tumours may lead to the failure of radiotherapy. In addition to their ability as radiosensitizers, nitroimidazoles are selectively cytotoxic to hypoxic cells (Adams *et al.*, 1978). The basis of structure–activity relationships in the development of electron-affinic nitroheterocyclic hypoxic cell radiosensitizers is a linear correlation of the type:

$$—\log C = b_0 + b_1 E + b_2 \log P + b_3 (\log P)^2$$

where C is the drug concentration required to cause a specific and relevant biological effect, E is the electron affinity, usually expressed as the one-electron redox potential ($E_7{}^1$), and P is the lipid-water partition coefficient of the drug. Adams *et al.* (1979*a,b*) have shown that lipophilicity has a negligible effect on radiosensitizing efficiency and cytotoxicity. Thus coefficients $b_2$ and $b_3$ may be omitted, yielding the simplified equation

$$—\log C = b_0 + b_1 E$$

It is well-established that the $E_7{}^1$ value correlates positively with radiosensitization efficiency (Adams *et al.*, 1976, 1979*a*), aerobic cytotoxicity (Adams *et al.*, 1979*b*), mutagenicity (Chin *et al.*, 1978) and hypoxic cytotoxicity (Adams *et al.*, 1980). The more electron-affinic the drug (the more positive the $E_7{}^1$ value) the greater the radiosensitization and cytotoxicity, which varies in general by an order of magnitude for each 100mV change in $E_7{}^1$.

These correlations suggest that redox processes are involved both in radiosensitization and cytotoxicity, but do not indicate a common mechanism, since radiosensitization is a fast process occurring in a few milliseconds (Adams *et al.*, 1975) and is temperature-independent, whereas cytotoxicity is relatively slow and is temperature-dependent (Stratford & Adams, 1977; Hall *et al.*, 1977). These criteria of nitroimidazole cytotoxicity to mammalian cells also apply to their effect on anaerobic microorganisms. However, the correlation of cytotoxicity and electron affinity is, in this case, a negative one: that is, the *less* electron-affinic the drug the greater its cytotoxicity, which generally doubles for each 100mV *decrease* in $E_7{}^1$ (Reynolds, 1980, 1981). The evidence that toxicities to hypoxic mammalian cells and to anaerobic microorganisms depend upon the reduction of the nitro group (Edwards *et al.*, 1973; Flockhart *et al.*, 1978) suggests a common mechanism.

To clarify the interaction of reduced nitroimidazoles with their target, we have developed an electrochemical model in which the nitro group of the drug may be selectively reduced at a controlled potential in the presence of DNA, and damage to the latter subsequently analysed (Knight *et al.*, 1978, 1979; Rowley *et al.*, 1979; Edwards *et al.*, 1980*a,b*). It has been established recently that reduced nitroimidazole-induced damage to DNA is related to its base composition (Rowley *et al.*, 1980) and is associated specifically with the release of thymidine phosphates from DNA (Knox *et al.*, 1980, 1981; Edwards *et al.*, 1980*b*). We report the use of such an *in vitro* model system to investigate structure–activity correlations of the reduced nitroimidazole drug–target interaction.

### MATERIALS AND METHODS

DNA type VIII from *Escherichia coli* B was obtained from the Sigma Chemical Co. Ltd, Dorset, and [$^{14}$C-C2]dT-labelled *E. coli* DNA from the Radiochemical Centre, Amersham, Bucks. Misonidazole (2-nitro-1-imidazolyl-3-methoxy-2-propanol), ornidazole (1-(3-chloro-2-hydroxypropyl)-2-methyl-5-nitroimidazole) and benznidazole (N-benzyl-1-(2-nitro-1-imidazolyl)acetamide) were generously donated by Roche Products Ltd, Welwyn Garden City, Herts, and metronidazole (1-2′-hydroxyethyl-2-methyl-5-nitroimidazole), dimetridazole (1,2-dimethyl-5-nitroimidazole) and 8609 RP (1,2-dimethyl-4-nitroimidazole) were generous gifts from May and Baker Ltd, Dagenham, Essex. Tinidazole (ethyl-1-[2-(2-methyl-5-nitroimidazolyl)-ethyl]sulphone) was donated by Pfizer Ltd, Sandwich, Kent. Nimorazole (4[2-(5-nitroimidazol-1-yl)ethyl]-morpholine) from Carlo Erba Ltd, Rome, Italy; azomycin (2-nitroimidazole) from the Sigma Chemical Co. and 4,(5)-nitroimidazole from the Aldrich Chemical Co., Gillingham, Dorset.

Reduction of the nitro group of each drug was carried out in the presence of DNA at potentials shown in the Table, as previously described (Knight *et al.*, 1979). In general, 10 mg *E. coli* DNA, 10 μg $^{14}$C-DNA and 20 μmoles of drug in 67 ml 15mM NaCl, 1·5mM trisodium citrate buffer, pH 7·1 (0·1 SSC) was made anoxic by purging with $N_2$ and reduction carried out using an Hg pool cathode and Ag/AgCl anode at an initial current density of 30 μA. Samples were removed before and after reduction, which was measured as the loss of absorbance at the λ max of each drug and zero current when reduction was completed. DNA damage was measured as the amount of [$^{14}$C]-dT release after dialysis for 18 h against water (Knox *et al.*, 1981). Alternatively, reduction was carried out at a constant potential of −500 mV for 24 h and DNA damage assessed as described above. The amount of reduction was measured spectrophotometrically as the relative decrease in the λ max of each drug.

Spectrophotometry was performed with a Pye-Unicam SP-800 Series B or SP 8150 scanning spectrophotometer, and radioactivity measured in a liquid scintillation spectrometer as previously described (Knox *et al.*, 1981).

All polarographic half-wave potentials ($E\frac{1}{2}$) were determined as previously described (Knight *et al.*, 1979) and $E\frac{1}{2}$ values quoted are those relative to the standard Ag/AgCl electrode. Values of the one-electron redox potential ($E_7{}^1$) are taken from published data and are relative to the normal hydrogen electrode.

All data points relating to log $^1/C$ are accurate to ±5% of any quoted value, and the straight-line and correlation data are computer-derived using a least-squares programme.

### RESULTS

The Table summarizes the results from 10 nitroimidazoles reduced in the presence of *E. coli* DNA, where damage is measured as the percentage total dT released by the action of the reduced drug. The results were fitted to a Hansch-type plot (Fig. 1) which shows a linear correlation described by the equation, and corresponds to that obtained by Reynolds (1981) in *Bacteriodes fragilis*, where cytotoxicity was assessed by minimum inhibitory concentration.

$$—\log C = -0{\cdot}003\, E_7{}^1 - 1{\cdot}4 \quad (r = 0{\cdot}70)$$

A similar, negative, correlation is obtained if $E\frac{1}{2}$ values are used in place of the

TABLE.—*Redox values of and damage produced by reduced nitroimidazoles*

| No. | Drug | $E\frac{1}{2}$ | $E_7^1$ | Damage | Log 1/c† | Reduction potential (mV) |
|---|---|---|---|---|---|---|
| 1 | Benznidazole | −200 | −380 | 2·7 | −0·569 | −700 |
| 2 | Misonidazole | −272 | −389 | 5·0 | −0·301 | −800 |
| 3 | Nimorazole | −345 | −457 | 4·05 | −0·393 | −850 |
| 4 | Ornidazole | −345 | −467 | 7·4 | −0·131 | −850 |
| 5 | Tinidazole | −340 | −464 | 12·7 | 0·104 | −850 |
| 6 | Azomycin | −374 | −418 | 6·0 | −0·222 | −900 |
| 7 | Metronidazole | −382 | −486 | 9·5 | −0·022 | −900 |
| 8 | Dimetridazole | −388 | −475 | 10·4 | 0·017 | −900 |
| 9 | 8609 RP | −475 | −550* | 10·3 | 0·013 | −1000 |
| 10 | 4(5) Nitroimidazole | −540 | −527 | 7·5 | −0·125 | −1000 |

$E\frac{1}{2}$ is the polarographic half-wave potential in mV measured against an Ag/AgCl reference electrode at pH 7·0.

$E_7^1$ is the one-electron redox potential in mV measured against the normal hydrogen electrode.

Damage measured as the percentage release of [$^{14}$C]-dT from DNA.

† C is the calculated drug-nucleotide ratio to produce a 10% release of dT from DNA.

* The value is computer-calculated on the basis of structural similarities to other drugs (Wardman, personal communication).

FIG. 1.—Linear correlation between DNA damage and electron affinity for 10 nitroimidazoles. (Identification in Table.) Log 1/c and $E_7^1$ are defined in footnote to Table.

FIG. 2.—Relationship between DNA damage measured as the percentage dT release and the electron affinity of 4 nitroimidazoles. (See Table.)

FIG. 3.—Relationship between relative reduction of nitroimidazoles and their electron affinity.

$E_7^1$ values. As partition effects play no part in the electrochemical model, no attempt has been made to include them, since they play an insignificant part in mammalian cells (Adams *et al.*, 1979*a,b*, 1980), bacteria (Reynolds, 1981) or protozoa (Chien & Mizuba, 1978).

An alternative mode of electrolytic reduction was carried out on 4 nitroimidazoles covering the range ($E\frac{1}{2}$) of −272 to −475 mV which were reduced at −500 mV for 24 h. This experiment was designed to model weak redox systems, which probably correspond to those in hypoxic cells. The results of such reductions when plotted graphically, show a linear relationship between the log of DNA damage and the electron affinity (Fig. 2) which is described by the equation:

$$-\log C = 0{\cdot}011\ E_7{}^1 + 4{\cdot}474\ (r = 0{\cdot}97)$$

This correlation is very similar to those obtained between electron affinity and cytotoxicity in hypoxic cells (Adams *et al.*, 1980; Olive, 1979*b*, 1980).

This latter correlation, however, arises as a consequence of the amount of drug reduced, as may be seen from Fig. 3, which shows an identical correlation between the log of drug reduction and their electron affinity. In all the results described it is significant that the unreduced drugs show none of these effects.

### DISCUSSION

The results establish that the correlation of drug-induced damage and electron affinity is related to the amount of drug reduced, and thus depends upon the end-point chosen to assess the cytotoxic effect of any drug.

If complete drug reduction occurs, a negative correlation is obtained (Fig. 1) which is almost identical to that found for anaerobic bacteria (Reynolds, 1981). However, if a unit timescale is considered, a positive correlation is obtained, identical to that found in hypoxic cells as indicated by cell survival, inhibition of cell growth, mutagenicity, inhibition of DNA synthesis or production of DNA strand breaks (Adams *et al.*, 1979*b*; Olive, 1979*b*, 1980). Since the mechanism of action of cytotoxicity is identical in each case (*viz.* DNA damage) it becomes apparent that a positive correlation arises as a result of the different rates of reduction of the drugs, which are themselves a direct function of their relative electron affinity (Fig. 3). The metabolic reduction rates of nitroimidazoles in hypoxic cells are well established (Olive, 1979*a*, 1980) and show an identical correlation with electron affinity to those relative rates obtained by electrolytic reduction at constant potential. Thus from published data (Olive, 1979*a*, 1980) the positive correlation obtained for $E_7{}^1$ and cytotoxicity in hypoxic mammalian cells can be corrected for relative drug-reduction rates to produce a negative correlation.

The $E_7{}^1$ value is a measure of the electron affinity of the nitro group which determines radiosensitization properties (Adams *et al.*, 1976; 1979*a*). However, the anaerobic cytotoxicity is generally considered to be due to a reduced species, the concentration of which is governed by the rate of its formation (*i.e.* reduction) which depends upon a reduction-rate-generated concentration gradient (Ings *et al.*, 1974) and its relative stability. Since the data shown in Fig. 1 do not involve reduction rates or concentration gradients, the results indicate that the reduced drug derivative is more stable at low $E_7{}^1$ values than at high ones, and the correlation in Fig. 1 may well reflect the relative stabilities of the cytotoxic agents. Experiments to determine the relative stabilities of reduced one-electron derivatives of nitroimidazoles are in progress.

Although the present study is limited to *E. coli*, previous studies have shown that specific dT release occurs from DNAs of *Micrococcus lysodeikticus*, *E. coli*, calf thymus and *Clostridium perfringens*; *i.e.* with A + T values ranging from 28% to 71%. In addition, whilst maximum release occurs from poly(d[AT]) none occurs from poly(d[GC]) (Rowley *et al.*, 1980; Knox *et al.*, 1980, 1981). This suggests that although the magnitude of the cytotoxicity may vary with the DNA A + T content of the cell, the results obtained in the present study would be applicable to all cell types.

The results support the hypothesis that the cytotoxic mechanism of action of reduced nitroimidazoles is identical in hypoxic mammalian cells, bacteria and protozoa. Differences would arise, however, in hypoxic cells due to the weak redox systems which predominate, resulting in those drugs which are cytotoxically potent in anaerobes being relatively less effective in hypoxic mammalian cells *in vivo*.

Although radiosensitization and cytotoxicity of nitroheterocyclic drugs *in vivo* may both be readily predicted from their electron affinities, these effects are, however, mechanistically distinct.

We thank the Cancer Research Campaign and the Medical Research Council for generous support, and Professor G. E. Adams for helpful discussion. R.C.K. is a Cancer Research Campaign Research Fellow and R.J.K. a Cancer Research Campaign postgraduate research assistant.

### REFERENCES

ADAMS, G. E., MICHAEL, B. D., ASQUITH, J. C., SHENOY, M. A., WATTS, M. E. & WHILLANS, D. W. (1975) Rapid mixing studies on the timescale of radiation damage in cells. In *Radiation Research: Biomedical, Chemical and Physical Perspectives*. Ed. Nygaard *et al.* London: Academic Press. p. 478.

ADAMS, G. E., FLOCKHART, I. R., SMITHEN, C. E., STRATFORD, I. J., WARDMAN, P. & WATTS, M. E. (1976) Electron-affinic sensitization. VII. A correlation between structures, one-electron reduction potentials, and efficiencies of nitroimidazoles as hypoxic cell radiosensitizers. *Radiat. Res.*, **67**, 9.

ADAMS, G. E., FOWLER, J. F. & WARDMAN, P. (Eds) (1978) Hypoxic cell sensitizers in radiobiology and radiotherapy. *Br. J. Cancer*, **37** (Suppl. III).

ADAMS, G. E., CLARKE, E. D., FLOCKHART, I. R. & 5 others (1979*a*) Structure–activity relationships in the development of hypoxic cell radiosensitizers. I. Sensitization efficiency. *Int. J. Radiat. Biol.*, **35**, 133.

ADAMS, G. E., CLARKE, E. D., GRAY, P. & 4 others (1979*b*) Structure–activity relationships in the development of hypoxic cell radiosensitizers. II. Cytotoxicity and therapeutic ratio. *Int. J. Radiat. Biol.*, **35**, 151.

ADAMS, G. E., STRATFORD, I. J., WALLACE, R. G., WARDMAN, P. & WATTS, M. E. (1980) The toxicity of nitro compounds towards hypoxic mammalian cells *in vitro*: Dependence upon reduction potential. *J. Natl Cancer Inst.*, **64**, 555.

CHIEN, Y. W. & MIZUBA, S. A. (1978) Activity–electroreduction relationship of antimicrobial metronidazole analogues. *J. Med. Chem.*, **21**, 374.

CHIN, J. B., SHEININ, D. M. K. & RAUTH, A. M. (1978) Screening for the mutagenicity of nitro-group containing hypoxic cell radiosensitizers using *Salmonella typhimurium* strains TA100 and TA96. *Mutat. Res.*, **58**, 1.

EDWARDS, D. I., DYE, M. & CARNE, H. (1973) The selective toxicity of antimicrobial nitroheterocyclic drugs. *J. Gen. Microbiol.*, **76**, 135.

EDWARDS, D. I., ROWLEY, D. A., KNOX, R. J., SKOLIMOWSKI, I. M. & KNIGHT, R. C. (1980*a*) Nature of DNA damage induced by electrolytically reduced nitroimidazole drugs. In *Current Chemotherapy and Infectious Disease*. Ed. Nelson & Grassi. Washington: Am. Soc. Microbiol. p. 561.

EDWARDS, D. I., KNOX, R. J., ROWLEY, D. A., SKOLIMOWSKI, I. M. & KNIGHT, R. C. (1980*b*) The biochemistry of nitroimidazole drug action. In *The Host Invader Interplay*. Ed. Van den Bossche. Amsterdam: Elsevier/North Holland. p. 673.

FLOCKHART, I. R., LARGE, P., MALCOLM, S. L., MARTIN, T. R. & TROUP, D. (1978) Pharmacokinetic and metabolic studies of the hypoxic cell radiosensitizer misonidazole. *Xenobiotica*, **8**, 97.

HALL, E. J., ASTER, M., GEARD, C. & BIAGLOW, J. (1977) On the cytotoxicity of the hypoxic cell radiosensitizer Ro 07-0582. The effect of hyperthermia and the reversal of the cytotoxic effect with cysteamine. *Br. J. Cancer*, **35**, 809.

INGS, R. M. J., MCFADZEAN, J. A. & ORMEROD, W. E. (1974) The mode of action of metronidazole in *Trichomomas vaginalis* and other micro-organisms. *Biochem. Pharmacol.*, **23**, 1421.

KNIGHT, R. C., SKOLIMOWSKI, I. M. & EDWARDS, D. I. (1978) The interaction of reduced metronidazole with DNA. *Biochem. Pharmacol.*, **27**, 2089.

KNIGHT, R. C., ROWLEY, D. A., SKOLIMOWSKI, I. M. & EDWARDS, D. I. (1979) Mechanism of action of nitroimidazole antimicrobial and antitumour radiosensitizing drugs: Effect of reduced misonidazole on DNA. *Int. J. Radiat. Biol.*, **36**, 367.

KNOX, R. J., KNIGHT, R. C. & EDWARDS, D. I. (1981) Misonidazole-induced thymidine release from DNA. *Biochem. Pharmacol.*, **30**, 1925.

KNOX, R. J., KNIGHT, R. C. & EDWARDS, D. I. (1980) Mechanism of action of misonidazole. *ICRS Med. Sci. Biochem.*, **8**, 190.

OLIVE, P. L. (1979*a*) Correlation between metabolic reduction rates and electron affinity of nitroheterocycles. *Cancer Res.*, **39**, 4512.

OLIVE, P. L. (1979*b*) Inhibition of DNA synthesis by nitroheterocycles. I. Correlation with half-wave reduction potential. *Br. J. Cancer*, **40**, 89.

OLIVE, P. L. (1980) Mechanisms of the *in vitro* toxicity of nitroheterocycles, including Flagyl and misonidazole. In *Radiation Sensitizers*. Ed. Brady. U.S.A.: Masson Publ. p. 39.

REYNOLDS, A. V. (1980) Activity of nitrocompounds against strains of *E. coli* deficient in DNA repair. *J. Pharm. Pharmacol.*, **32**, 35p.

REYNOLDS, A. V. (1981) The activity of nitrocompounds against *Bacteroides fragilis* is related to their electron affinity. *J. Antimicrobial Chemother.*, **8**, 91.

ROWLEY, D. A., KNIGHT, R. C., SKOLIMOWSKI, I. M. & EDWARDS, D. I. (1979) The effects of nitroheterocyclic drugs on DNA: An *in vitro* model of cytotoxicity. *Biochem. Pharmacol.*, **28**, 3009.

ROWLEY, D. A., KNIGHT, R. C., SKOLIMOWSKI, I. M. & EDWARDS, D. I. (1980) The relationship between misonidazole cytotoxicity and base composition of DNA. *Biochem. Pharmacol.*, **29**, 2095.

STRATFORD, I. J. & ADAMS, G. E. (1977) The effect of hyperthermia on differential cytotoxicity of a hypoxic cell radiosensitizer Ro-07-0582 on mammalian cells *in vitro*. *Br. J. Cancer*, **35**, 307.